# Evaluation of cognitive and psychomotor faculties in relation to mood-related symptoms under the conditions of sleep deprivation

**DOI:** 10.3389/fpsyt.2023.1332831

**Published:** 2023-12-22

**Authors:** Marcin Sochal, Marta Ditmer, Piotr Białasiewicz, Szymon Turkiewicz, Filip Franciszek Karuga, Agata Gabryelska

**Affiliations:** Department of Sleep Medicine and Metabolic Disorders, Medical University of Lodz, Lodz, Poland

**Keywords:** sleep deprivation, PVT, hand-eye coordination, stroop, sleep

## Abstract

**Introduction:**

Deprivation of sleep (DS) has been associated with changes in mood and cognitive function, rapidly but transiently improving the severity of depression symptoms. However, it remains unclear whether there are differences in performance between DS responders and non-responders. The relationship between DS, mood, cognitive, and psychomotor function is also poorly understood.

**Methods:**

Participants (*n* = 77) underwent a baseline assessment of sleep under the control of polysomnography (PSG). Later they were subjected to DS with actigraphy monitoring. Evaluation of mood as well as completing a battery of tests assessing cognitive functions and eye-hand coordination was conducted four times, pre/post PSG and DS. Participants were further divided into respondents (RE, *n* = 48) and non-respondents (NR, *n* = 29) depending on alleviation of depression symptoms severity following DS.

**Results:**

All participants exhibited increased response speed to visual triggers after DS compared to baseline (*p* = 0.024). Psychomotor vigilance test (PVT) results remained intact in the RE, whereas it was increased in the NR (*p* = 0.008). Exposure time in the eye-hand coordination test improved in both groups, but total error duration was reduced only in RE individuals (*p* < 0.001, *p* = 0.009 for RE and NR, respectively). All subjects were more proficient at trail-making test (*p* ≤ 0.001 for Part 1 and 2 in all, NR, RE). Stroop test also improved regardless of mood changes after DS (*p* = 0.007, *p* = 0.008 for Part 1 and 2, respectively); cognitive interference remained at a similar level within groups (*p* = 0.059, *p* = 0.057 for NR and RE, respectively). A positive correlation was observed between the difference in PSG morning/DS morning depression severity and vigilance (*R* = 0.37, *p* = 0.001, *R* = 0.33, *p* = 0.005, for error duration eye-hand coordination test and PVT total average score, respectively).

**Conclusion:**

RE tend to maintain or improve cognitive function after DS, oppositely to NR. Vigilance in particular might be tightly associated with changes in depression symptoms after DS. Future studies should examine the biological basis behind the response to sleep loss.

## 1 Introduction

Deprivation of sleep (DS) is a term used to describe the forced elimination of sleep, either externally (e.g., work, noise) or internally (e.g., pain). It should not be confused with insomnia, which is characterized by an inability to fall asleep despite the right conditions. Studies conducted up to date strongly suggest that quality of life and sleep (or rather a lack thereof) are tightly connected. In a study by Seweryn et al. on patients with temporomandibular joint disorders, more than 50% reported poor sleep quality, which was associated with temporal muscle pain and average muscle pain (1). Evidence suggests that impaired sleep (in the discussed case caused by sleep deprivation due to pain), could in turn further aggravate pain symptoms, creating a vicious circle (1). Moreover, the authors showed that sleep quality had a greater impact on life quality than contrariwise (1). Interestingly, in the case of migraines, a neurological disorder characterized primarily by headaches, both sleep deprivation and excessive sleep were connected to morning migraine attacks (2). In this group of patients, sleep disorders were also shown to be associated with poorer quality of life (2). As Hu and Wang demonstrated in their review, targeting sleep disturbances might significantly benefit patients with pain disorders (3).

Sleep loss can have differing effects on mood. While it has been shown to have a predominantly negative effect in healthy individuals, in the case of depression, DS is known for its rapid and transient antidepressant effect, which has been studied for a long time as a potential therapeutic measure. The improvement in mood after DS is described as a “response” and occurs in approximately 50–80% of depressed individuals (4). DS-induced mood enhancement might also be present in younger individuals without affective disorders (5–8). The biological basis for such impact of DS is elusive; it is suspected that neurotrophin surge following acute sleep loss might play an important role (9).

It also remains underexplored how different types of response to sleep loss reflect themselves in other aspects of functioning. Studies conducted up to date on the general population show that DS might exert detrimental effects on cognition, particularly strongly affecting non-executive functions (10, 11). In contrast, tasks requiring complex attention and working memory appear to be at most only moderately disturbed by DS (11). It is thought that simple tasks, such as the psychomotor vigilance test (PVT), which measures response speed, are mostly based on attention and involve fewer processing neural areas. Therefore, their results are particularly susceptible to being skewed by fatigue (12).

Much less is known about this subject in the context of depression as well as the relationship between mood and discussed abilities in general. Lu et al. in their small study group (18 depressed patients) demonstrated that the executive function of patients with depression might improve after total DS, but participants were not divided into groups based on response to sleep loss (13). In a study by Larson et al. depressed individuals exhibited cognitive decrement similar to that seen in the control group after sleep loss; mood was not studied (14).

Therefore, the aim of the present study was to compare cognitive and psychomotor faculties after PSG and DS, in relation to alterations in the severity of depression symptoms.

## 2 Materials and methods

Participants (*n* = 113) were recruited at the Department of Sleep Medicine and Metabolic Disorders, Medical University of Lodz. Out of the group, 31 individuals did not finish the protocol. The data of 82 participants were subject to analysis.

Inclusion criteria comprised: informed consent forms for polysomnography (PSG) and DS, adherence to the protocol, age 18–35, body mass index 20–30 kg/m^2^.

Exclusion criteria encompassed: chronic disease (neurological, psychiatric, endocrine, metabolic, inflammatory, circulatory/renal/respiratory insufficiency), neoplasms (except for basal-cell carcinoma), history of surgery within the last 6 months, history of radio/chemotherapy, pregnancy/breastfeeding, substance abuse, use of medications potentially affecting sleep (hypnotics, steroids, neuroleptic, anxiolytics, antidepressants, etc.), intercontinental flight 2 weeks prior/during the study protocol, sleep disorders.

**Figure 1:**
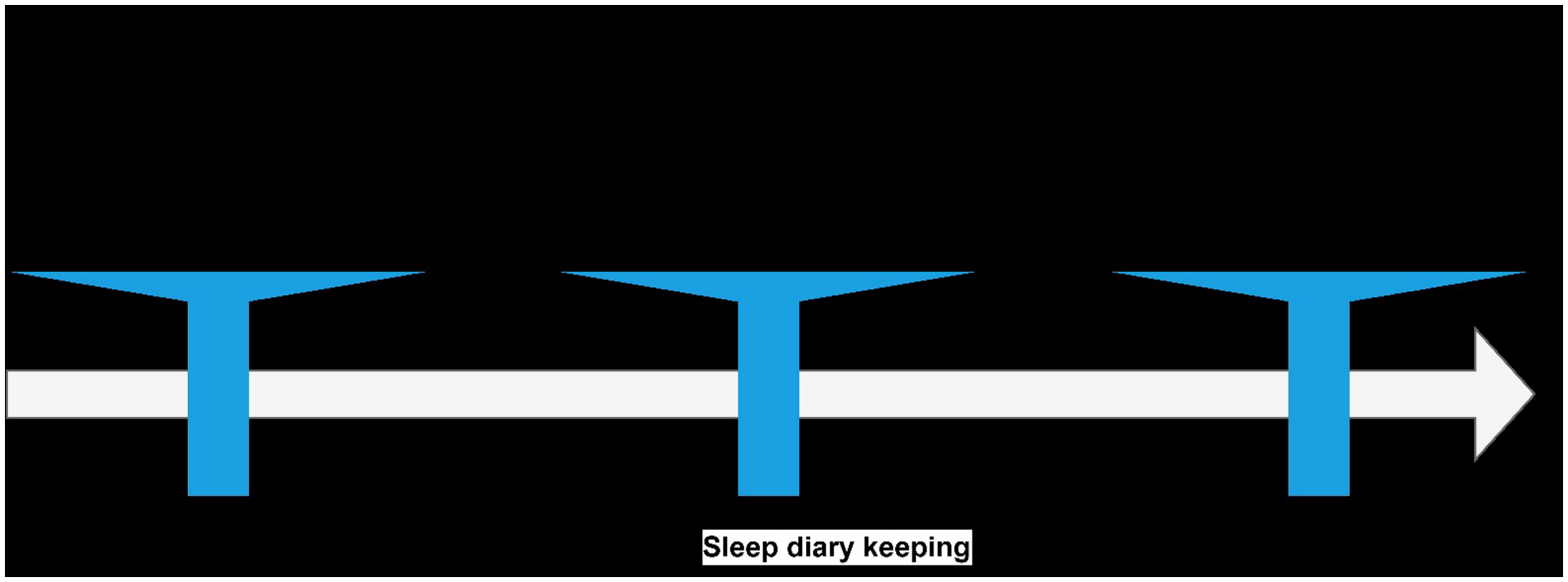
Study methodology. Questionnaires, scales and tests presented in the figure assessing depression symptoms (MADRS) and psychomotor functioning (PVT, TMT, The Stroop and Word Test, BEHCT). BEHCT, Bimanual Eye-Hand Coordination Test; DS, sleep deprivation; MADRS, Montgomery–Åsberg Depression Rating Scale; PSG, polysomnography examination; PVT, Psychomotor vigilance test; TMT, Trail Making Test.

The study protocol consisted of two phases: PSG and DS. The study methodology was presented in the Figure 1.

Participants were asked to keep a sleep diary for 2 weeks before the PSG examination. The researchers provided a template, which included information about naps, time spent in bed, estimated sleep time, sleep quality, number and duration of awakenings, and sleep quality.

In the evening of the PSG day, participants were admitted to the Department at *ca.*7 PM. After the interview and general physical examination PSG would begin around 10 PM and end at 7 AM on the following morning.

Parameters assessed during the examination comprised: electromyography, electrocardiography (precordial leads V1–V2), electroencephalography, electrooculography, airflow and respiratory effort signals (chest, abdomen), SpO2 saturation, and body position. PSG was performed with the use of Alice 4, Phillips-Respironics (USA).

Interpretation of the recordings were performed by the same researcher, in line with the criteria set by the American Academy of Sleep Medicine, using a standard 30-s epoch length (15).

Subjects with average sleep time < 5 h were excluded from the analysis.

Twenty-four-hour DS took place between 2 and 4 weeks after PSG. Participants were allowed to spend the night at their will, provided it was in compliance with the protocol. Similarly to PSG, they were obliged to visit the Department in the evening and the next morning.

Actigraphy was used in order to monitor the physical activity of enrolled individuals. Actigraphs (actigraph GENEActive Original, ActivInsights Ltd.) were distributed among the participants on the evening of the DS day. Scoring and interpretation of actigraphy were performed by the same person for the sake of standardization.

Questionnaires and scales assessing depression symptoms as well as tests of cognitive and psychomotor functions, were performed before and after the PSG and repeated on the DS day in the same fashion.

*Montgomery–Åsberg Depression Rating Scale (MADRS)* is a 10-item scale assessing symptoms of depression. It includes domains like sadness, tension, and sleep. An outcome above 7 indicates mild depression. Participants were classified as Non-Respondents (NR; without overnight improvement in MADRS score) or Respondents (RE; improvement in MADRS score or score < 8 which remained the same overnight).

*Psychomotor vigilance test* (PVT, Response Time Test Apparatus, AT Smart Systems, Poland) is a test assessing eye-hand coordination and reaction speed. The subject is supposed to press a button on a joystick as soon as they register a visual or auditory signal.

*Trail Making Test (TMT)* is an instrument used in the assessment of executive function, visual-motor faculties, processing skills, etc. (16). A subject is given a sheet with either a set of dispersed numbers (part A) or numbers and letters (part B) (16). An individual is supposed to connect elements in an ascending order in the shortest possible amount of time (16).

*The Stroop Color and Word Test* is a tool used to evaluate executive functions and the ability to suppress cognitive dissonance (17). The subject is instructed to read aloud 25 words; in the first part of the test color of the word does not match its meaning, and in the second part there is no such discrepancy (17).

*Bimanual Eye-Hand Coordination Test (BEHCT)* is a test used to measure eye-hand coordination. An individual is supposed to draw over a star contour using a stylus. The stylus is controlled by two handwheels, for vertical and horizontal movement. The instrument measures the total time spent on the task, the number of errors (crossing over the line), and the amount of time spent in an incorrect position.

The study protocol was approved by The Bioethical Committee of the Medical University of Lodz (number: RNN/302/20/KE).

Data analysis was performed using Statistica 13.1 PL (StatSoft, Tulsa, OK, USA). P below 0.05 was considered statistically significant. For continuous variables, the evaluation of distribution (normal/non-normal) was conducted with the Shapiro–Wilk test. For the normal distribution, data were presented as mean with standard deviation (SD); if the distribution was non-normal, median with interquartile range (IQR: first–third quartile) was applied. The t-student test was used for parametric independent variables. Nonparametric dependent or independent variables were analyzed with Wilcoxon signed-rank or Mann–Whitney U test, respectively. Correlations were assessed with Spearman’s correlation test.

## 3 Results

Demographic characteristics of participants (*n* = 77) were provided in Table 1. Based on the MADRS score, individuals were assigned to one of the following groups: Non-Respondents (NR; *n* = 29) or Respondents (RE; *n* = 48); the response rate reached 62%. There were no statistically significant differences in the aforementioned parameters between the groups (all *p* > 0.05; Table 1).

**Table 1:** Baseline characteristics of study participants.

Parameter	Non-respondents	Respondents	*p*
*n*	29	48	–
Women, *n*, %	15, 51.70%	23, 47.90%	0.883
Age, median (IQR)	23 (22–26)	24 (22–26)	0.541
BMI, [kg/m2], median (IQR)	22.05 (19.49–23.78)	22.85 (22.04–24.72)	0.065
Smoking, *n*, %	3, 10.30%	6, 12.50%	0.775
Higher education, *n*, %	14, 48.30%	23, 47.90%	0.735
Total sleep time during the PSG night, [min], median (IQR)	400 (360–480.5)	430 (366–468.75)	0.987
Sleep latency during the PSG night, [min], median (IQR)	33.5 (17.5–51)	34.5 (23.75–57.75)	0.467
Sleep efficiency during the PSG night [%], median (IQR)	78.6 (71.7–89.5)	81 (69.25–85.65)	0.487

BMI, Body Mass Index; PSG, polysomnography. Results were presented as median (Interquartile range, IQR) or *n*, %.

Baseline PSG PVT scores were similar in comparison between NR and RE participants (*p* = 0.642, *p* = 0.317, *p* = 0.806, *p* = 0.493, *p* = 0.583, *p* = 0.480 for PVT total, acoustic, optic average in the evening and morning, respectively). Responsiveness to auditory stimuli was significantly improved in RE individuals in the morning after PSG compared to the preceding evening (*p* = 0.043, *p* = 0.002, and for PVT total and auditory average, respectively; Table 2). NR subjects did not exhibit better reaction time to triggers. A correlation between differences in depression severity, total and acoustic PVT average at discussed time points was also noted (*R* = 0.26, *p* = 0.023; *R* = 0.31, *p* = 0.006, respectively; Table 3). Both groups made progress regarding BEHCT, improving their exposure time, number, and duration of errors (*p* < 0.001, *p* = 0.031, *p* < 0.001; *p* < 0.001, *p* = 0.003, *p* < 0.001, for NR and RE, respectively; Table 2). After a night’s rest, all participants were more proficient in Part and in Part 2 of the Stroop test (all *p* < 0.001; Table 2). Similar improvements were noted regarding TMT Part 1 (*p* < 0.001, *p* = 0.002; *p* = 0.012, *p* < 0.001 for NR and RE for Part 1 and 2, respectively; Table 2).

**Table 2:** Results of cognitive and psychomotor tests of non-depressed and at least mildly depressed participants in the evening of the polysomnography day and on the following morning.

	All			Non-respondents			Depression		
	PSG evening n; median IQR	PSG morning n; median IQR	*p*	PSG evening n; median IQR	PSG morning n; median IQR	*p*	PSG evening n; median IQR	PSG morning n; median IQR	*p*
PVT total average [s]	77; 0.25 (0.23–0.27)	77; 0.24 (0.23–0.26)	**0.036**	29; 0.24 (0.23–0.27)	29; 0.25 (0.23–0.27)	0.417	48; 0.25 (0.23–0.27)	48; 0.24 (0.22–0.26)	**0.043**
PVT acoustic average [s]	77; 0.24 (0.2–0.29)	77; 0.22 (0.20–0.26)	0.657	29; 0.23 (0.21–0.27)	29; 0.23 (0.2–0.26)	0.247	48; 0.25 (0.2–0.3)	48; 0.22 (0.19–0.26)	**0.002**
PVT optic average [s]	77; 0.25 (0.23–0.27)	77; 0.25 (0.24–0.26)	0.283	29; 0.25 (0.24–0.27)	29; 0.25 (0.24–0.28)	0.496	48; 0.25 (0.23–0.27)	48; 0.25 (0.23–0.26)	0.367
BEHCT exposure time [s]	74; 263.00 (223.00–343.00)	74; 234.50 (196.00–284.00)	**<0.001**	27; 272.00 (223.00–366.00)	27; 240.00 (199.00–300.00)	**<0.001**	47; 250.00 (216.00–327.00)	47; 234.00 (192.00–283.00)	**<0.001**
BEHCT error time [s]	74; 36.30 (24.80–53.10)	74; 24.25 (14.90–43.00)	**<0.001**	27; 35.70 (24.80–61.50)	27; 28.00 (11.40–45.50)	**0.031**	47; 36.90 (24.50–52.50)	47; 23.10 (15.70–42.70)	**0.003**
BEHCT number of errors	74; 28 (17–42)	74; 16 (10–25)	**<0.001**	27; 30 (17–55)	27; 17 (6–33)	**<0.001**	47; 26 (16–39)	47; 16 (10–23)	**<0.001**
Stroop test part 1 [s]	76; 12.62 (11.03–15.01)	77; 11.14 (9.98–12.47)	**<0.001**	29; 13.58 (11.4–16.08)	29; 11.50 (10.67–12.94)	**<0.001**	47; 11.94 (10.64–14.32)	48; 11.05 (9.93–11.78)	**<0.001**
Stroop test part 2 [s]	76; 11.05 (9.95–12.58)	76; 10.36 (9.53–11.42)	**<0.001**	29; 11.46 (10.56–13.09)	29; 10.60 (9.81–11.92)	**<0.001**	47; 10.94 (9.81–12.16)	47; 10.07 (9.51–11.03)	**<0.001**
TMT part 1 [s]	77; 20.79 (17.82–24.97)	77; 18.10 (14.95–22.21)	**<0.001**	29; 19.62 (17.07–24.93)	29; 18.10 (14.50–21.52)	**<0.001**	48; 20.81 (18.64–25.11)	48; 17.82 (15–24.57)	**0.012**
TMT part 2 [s]	77; 44.09 (35.61–55.77)	77; 37.00 (28.67–47.45)	**<0.001**	29; 45.55 (35.81–55.00)	29; 38.25 (30.14–47.45)	**0.002**	48; 41.95 (34.81–62.88)	48; 35.98 (28.64–46.86)	**<0.001**

DS, deprivation of sleep; BEHCT, bimanual eye-hand coordination test; PSG, polysomnography; PVT, psychomotor vigilance test; TMT, trail making test. Results were presented as n; median (Interquartile range, IQR). Bolded text indicates statistical significance.

**Table 3:** Correlations between differences in depression severity and results of cognitive and psychomotor tests of participants pre/post polysomnography or sleep deprivation.

	Δ MADRS PSG evening/morning			Δ MADRS DS evening/morning			Δ MADRS PSG morning/DS morning		
	*n*	*R*	*p*	*n*	*R*	*p*	*n*	*R*	*p*
PVT total average [s]	77	0.26	**0.023**	77	0.36	**0.001**	77	0.37	**0.001**
PVT acoustic average [s]	77	0.31	**0.006**	77	0.24	**0.039**	77	0.33	**0.003**
PVT optic average [s]	77	0.18	0.11	77	0.34	**0.002**	77	0.32	**0.005**
BEHCT exposure time [s]	74	0.04	0.73	77	−0.13	0.257	74	−0.16	0.176
BEHCT error time [s]	74	0.05	0.68	77	0.15	0.179	74	0.23	**0.044**
BEHCT number of errors	74	−0.03	0.817	77	0.13	0.269	74	0.12	0.323
Stroop test part 1 [s]	76	−0.02	0.875	76	0.15	0.21	76	0.14	0.218
Stroop test part 2 [s]	76	−0.05	0.656	76	0.08	0.517	75	−0.02	0.861
TMT part 1 [s]	77	0.19	0.105	76	0.08	0.517	76	0.14	0.242
TMT part 2 [s]	77	−0.04	0.728	76	0.01	0.965	76	0.03	0.830

BEHCT, bimanual eye-hand coordination test; DS, deprivation of sleep; PSG, polysomnography; PVT, psychomotor vigilance test; TMT, trail making test. Bolded text indicates statistical significance.

In the evening of the DS day, there were no differences in reaction time between NR and RE (*p* = 0.165, *p* = 0.963, *p* = 0.140 for PVT total, acoustic, and optic average, respectively). In the morning it was observed that RE individuals had a better reaction speed and exposure time in BEHCT (*p* = 0.003, *p* = 0.041; *p* = 0.006, *p* = 0.040 for total, acoustic, optic PVT average and BEHCT exposure time, respectively) than the NR group. DS significantly impaired responsiveness to stimuli within both groups (*p* = 0.002, *p* = 0.001, *p* = 0.003; *p* < 0.001, *p* = 0.017, *p* = 0.001 for PVT total, acoustic, and optic average in NR and RE, respectively; Table 4). A positive correlation between the difference in MADRS score and PVT outcome pre/post DS was noted as well (*R* = 0.36, *p* = 0.001, *R* = 0.24, *p* = 0.039, *R* = 0.34, *p* = 0.002 for PVT total, acoustic, and optic average, respectively; Table 3). As for the BEHCT, exposure time in the morning after DS was decreased within the RE, but not the NR group (*p* < 0.001, 0.060 for Re and NR, respectively; Table 4), without changes to the number or duration of errors. Results of the Stroop Test remained unaffected. Regarding TMT, results of both parts of the task became better after a sleepless night in comparison with those from the preceding evening within each group (*p* = 0.150, *p* = 0.393; *p* = 0.033, *p* = 0,003 for Part 1 and 2 in NR and RE, respectively; Table 4).

**Table 4:** Results of cognitive and psychomotor tests of non-depressed and at least mildly depressed participants in the evening of the sleep deprivation day and on the following morning.

	All			Non-respondents			Respondents		
	DS evening n; median IQR	DS morning n; median IQR	*p*	DS evening n; median IQR	DS morning n; median IQR	*p*	DS evening n; median IQR	DS morning n; median IQR	*p*
PVT total average [s]	77; 0.23 (0.22–0.25)	77; 0.25 (0.23–0.27)	**<0.001**	29; 0.24 (0.22–0.26)	29; 0.26 (0.24–0.29)	**0.002**	48; 0.23 (0.22–0.25)	48; 0.24 (0.23–0.26)	**<0.001**
PVT acoustic average [s]	77; 0.21 (0.18–0.24)	77; 0.23 (0.20–0.27)	**<0.001**	29; 0.21 (0.18–0.24)	29; 0.25 (0.21–0.28)	**0.001**	48; 0.21 (0.18–0.24)	48; 0.22 (0.20–0.25)	**0.017**
PVT optic average [s]	77; 0.24 (0.23–0.26)	77; 0.25 (0.24–0.27)	**<0.001**	29; 0.24 (0.23–0.26)	29; 0.26 (0.25–0.29)	**0.003**	48; 0.24 (0.22–0.26)	48; 0.24 (0.23–0.26)	**0.001**
BEHCT exposure time [s]	77; 201 (172–258)	77; 192 (156–230)	**<0.001**	29; 188 (162–267)	29; 200 (153–230)	0.060	48; 205.5 (181–248.5)	48; 185 (160–228)	**<0.001**
BEHCT error time [s]	77; 25.20 (15.30–34.70)	77; 20.30 (11.60–34.90)	0.162	29; 26.40 (15.20–42.10)	29; 26.80 (14.40–48.60)	0.905	48; 24.55 (15.55–32.25)	48; 19.45 (9–27.85)	0.091
BEHCT number of errors	77; 17 (12–25)	77; 15 (8–24)	0.598	29; 18 (10–28)	29; 19 (9–36)	0.313	48; 17 (12–21.5)	48; 14 (8–22)	0.153
Stroop test part 1 [s]	76; 10.87 (9.42–11.93)	76; 10.83 (9.79–11.75)	0.636	29; 10.72 (9.39–12.38)	29; 10.92 (10.04–11.92)	0.459	47; 10.94 (9.42–11.91)	47; 10.46 (9.73–11.54)	0.165
Stroop test part 2 [s]	76; 9.92 (9.10–11.17)	76; 10.06 (9.28–11.11)	0.989	29; 9.77 (9.25–11.30)	29; 10.30 (9.28–11.16)	0.927	47; 9.95 (9.08–10.94)	47; 9.92 (9.25–10.90)	0.982
TMT part 1 [s]	77; 16.04 (13.13–20.25)	76; 14.75 (12.44–18.52)	**0.010**	29; 15.23 (12.73–19.00)	29; 16.41 (12.30–18.54)	0.150	48; 16.47 (13.20–20.35)	47; 14.49 (12.57–18.35)	**0.033**
TMT part 2 [s]	77; 32.80 (25.42–39.99)	76; 28.42 (21.71–38.60)	**0.006**	29; 29.69 (25.42–35.44)	29; 29.61 (21.72–35.72)	0.393	48; 34.05 (25.51–41.24)	47; 28.00 (21.24–38.98)	**0.003**

BEHCT, bimanual eye-hand coordination test; DS, deprivation of sleep; PSG, polysomnography; PVT, psychomotor vigilance test; TMT, trail making test. Results were presented as *n*; median (Interquartile range, IQR). Bolded text indicates statistical significance.

On the between-group analysis of test results obtained in the morning after PSG and DS, RE had better PVT scores and BEHCT error time than NR (*p* = 0.003, *p* = 0.041, *p* = 0.006, *p* = 0.040 for PVT total, acoustic, optic average, and BEHCT error time respectively). Reaction speed to visual stimuli was impaired in all participants (*p* = 0.024) after the DS. PVT total and optic average was impaired in the NR group (*p* = 0.008, and *p* = 0.005 for PVT total, and optic average, respectively; Table 5); no changes were seen among RE subjects when comparing morning PSG vs. morning DS scores. The exposure time in BEHCT decreased between the discussed time points regardless of response to DS, without changes to error number or duration (*p* < 0.001 for both NR and RE; Table 5). A similar observation was made for TMT Part 1 and 2 (*p* < 0.001, *p* = 0.001; *p* < 0.001, *p* < 0.001 for Part 1 and 2 in NR and RE respectively; Table 5). All participants improved their scores in both parts of the Stroop test (*p* = 0.007, *p* = 0.008 for Part 1 and 2; Table 5). NR, but not RE exhibited improvement in Part 2 of the task (*p* = 0.021 for NR; Table 5) after DS when compared to the baseline PSG morning. There was a positive correlation between the difference in depression severity and PVT scores, as well as error time in BEHCT (*R* = 0.37, *p* = 0.001, *R* = 0.33, *p* = 0.005, *R* = 0.32, *p* = 0.005, *R* = 0.23, *p* = 0.044, for BEHCT error time, PVT total, acoustic, and optic average, respectively; Table 3).

**Table 5:** Results of cognitive and psychomotor tests of non-depressed and at least mildly depressed participants in the morning after polysomnographic examination or sleep deprivation.

	All			Non-respondents			Respondents		
	PSG morning n; median IQR	DS morning n; median IQR	*p*	PSG morning n; median IQR	DS morning n; median IQR	*p*	PSG morning n; median IQR	DS morning n; median IQR	*p*
PVT total average [s]	77; 0.24 (0.23–0.26)	77; 0.25 (0.23–0.27)	0.051	29; 0.25 (0.23–0.27)	29; 0.26 (0.24–0.29)	**0.008**	48; 0.24 (0.22–0.26)	48; 0.24 (0.23–0.26)	0.735
PVT acoustic average [s]	77; 0.22 (0.20–0.26)	77; 0.23 (0.20–0.27)	0.211	29; 0.23 (0.20–0.26)	29; 0.25 (0.21–0.28)	0.069	48; 0.22 (0.19–0.26)	48; 0.22 (0.20–0.25)	0.850
PVT optic average [s]	77; 0.25 (0.24–0.26)	77; 0.25 (0.24–0.27)	**0.024**	29; 0.25 (0.24–0.28)	29; 0.26 (0.25–0.29)	**0.005**	48; 0.25 (0.23–0.26)	48; 0.24 (0.23–0.26)	0.601
BEHCT exposure time [s]	74; 234.5 (196–284.00)	77; 192 (156–230)	**<0.001**	27; 240 (199–300)	29; 200 (153–230)	**<0.001**	47; 234 (192–283)	48; 185 (160–228)	**<0.001**
BEHCT error time [s]	74; 24.25 (14.9–43)	77; 20.3 (11.6–34.9)	0.081	27; 28 (11.4–45.5)	29; 26.8 (14.4–48.6)	0.524	47; 23.10 (15.70–42.70)	48; 19.45 (9.00–27.85)	**0.009**
BEHCT number of errors	74; 16 (10–25)	77; 15 (8–24)	0.239	27; 17 (6–33)	29; 19 (9–36)	0.525	47; 16 (10–23)	48; 14 (8–22)	0.052
Stroop test part 1 [s]	77; 11.14 (9.98–12.47)	76; 10.83 (9.79–11.75)	**0.007**	29; 11.50 (10.67–12.94)	29; 10.92 (10.04–11.92)	0.059	48; 11.05 (9.93–11.78)	47; 10.46 (9.73–11.54)	0.057
Stroop test part 2 [s]	76; 10.36 (9.53–11.42)	76; 10.06 (9.28–11.11)	**0.008**	29; 10.60 (9.81–11.92)	29; 10.30 (9.28–11.16)	**0.021**	47; 10.07 (9.51–11.03)	47; 9.92 (9.25–10.90)	0.129
TMT part 1 [s]	77; 18.10 (14.95–22.21)	76; 14.75 (12.44–18.52)	**<0.001**	29; 19.62 (17.07–24.93)	29; 16.41 (12.30–18.54)	**<0.001**	48; 17.82 (15.00–24.57)	47; 14.49 (12.57–18.35)	**<0.001**
TMT part 2 [s]	77; 37 (28.67–47.45)	76; 28.42 (21.71–38.6)	**<0.001**	29; 45.55 (35.81–55.00)	29; 29.61 (21.72–35.72)	**0.001**	48; 35.98 (28.64–46.86)	47; 28.00 (21.24–38.98)	**<0.001**

BEHCT, bimanual eye-hand coordination test; DS, deprivation of sleep; PSG, polysomnography; PVT, psychomotor vigilance test; TMT, trail making test. Results were presented as n; median (Interquartile range, IQR). Bolded text indicates statistical significance.

## 4 Discussion

Studies on the subject of DS usually focus either on the impairment of cognitive/psychomotor functions in subjects without affective disorders or changes in the severity of depression symptoms in already diagnosed individuals; combined assessment of mood and cognitive faculties is rarely conducted.

The response rate to DS was 62%, which is similar to the one seen in depressed patients, i.e., 50–80%; there are no universal criteria describing response to DS, which contributes to discrepancy in studies’ results (4). Studies on healthy subjects do not contain the response rate, however, Tomaso et al. in their meta-analysis noted, that sleep loss might exert a positive effect on negative mood, which is particularly strong in studies on younger individuals, thus fitting the description of the study group (8).

Studies on the subject of DS tend to compare post-DS results to the baseline values obtained at a similar part of the day, to exclude any interference caused by circadian rhythmicity. Here, a comparison of PSG morning vs. DS morning showed impaired responsiveness in all participants and within the NR group, but not within the RE group. All study subjects as well as both groups analyzed separately improved BEHCT exposure time and TMT, which could be ascribed to the learning effect. Additionally, RE exhibited better BEHCT error time than NR, indicating enhanced vigilance and executive functions. All study subjects were more proficient at the Stroop test at the end of the study than during the baseline assessment, however, within groups cognitive interference was not altered; NRs were better only at facilitation.

To the best of our knowledge, there are no studies assessing the cognitive abilities of DS RE vs. NR. Results of other projects investigating the consequences of sleep deprivation, conducted on the general population without dividing participants based on response to sleep loss match those obtained here. Philibert concluded in her meta-analysis that DS impaired primarily vigilance and sustained attention (the effect size was *d* = −1.33, 95% CI [−1.124, −1.536]), compared to the overall decrease in cognitive faculties (−0.564, 95% CI [−0.406, −0.722]); studies without baseline values or control group were excluded from the analysis (10). Other researchers noted similar results, with the average mean effect sizes of three meta-analyses being moderate, namely *d* = 0.72 (11, 18–21). There are also papers where no impairment of attention was noted, albeit in such cases a different battery of tests was applied (22–24). Literature assessing the influence of sleep deprivation on TMT or specific psychomotor capabilities is more limited. O’Hagan et al. in their project also found that hand-eye coordination is significantly impaired following sleep loss (25). Dawson et al. reported similar results after 17 h of constant wakefulness (26). According to a recent meta-analysis, psychomotor functions in general appear to be moderately stunted by DS, with the average effect size being 0.77 (19). In another experiment on college students, neither of the TMT parts was affected by sleep deprivation (27). A study by Fluck et al. showed similar results in junior residents (28). As for the Stroop test, Sagaspe et al. also did not observe any differences in interference control or accuracy after DS (29). Cain et al. conducted an experiment involving 40 h of constant wakefulness; they concluded that even though response time increased, interference or facilitation (abilities underlying performance in Part 1 and 2, respectively) did not alter after sleep loss (30). Binks et al. obtained similar results (24).

Discrepancies between the studies regarding absolute values, which further influence effect sizes might stem from differences in applied protocols or equipment. The learning effect is another important confounder for all tasks applied here, except for PVT (31, 32).

The compromising effect of DS on cognitive abilities might be explained by disturbances in the prefrontal cortex (PFC) function. Harris et al. in their study applied a battery of tests specifically targeting this brain region; after 36-h DS younger (19–27 y.o.) subjects had similar results to their older counterparts (55–64 y.o.) (33). Functional changes are also present in electroencephalography recordings. Verweij et al. have reported a decrease in alpha frequency and an increase in theta frequency (markers for local functional interconnectedness and global functional connectivity, respectively) in the prefrontal region (34). Dai et al. observed an increase in connectivity between default mode network and dorsal attention network, which could contribute to misplaced attention, focus pointed inward on the self rather than on the task at hand (35).

A significant positive correlation was noted between differences in mood (Δ MADRS) and PVT total and acoustic average at all time points, as well as BECT error time at the PSG morning/DS morning, suggesting that mood is linked to cognitive and psychomotor faculties. This is an interesting subject for future studies, as individuals with mood disorders like depression frequently experience psychomotor retardation and score lower on PVT than their healthy counterparts (36–38). Stroop test results, but not TMT are also lower, albeit only in clinically depressed patients-studies comparing dysphoric individuals with healthy controls showed no differences (39, 40). Even though DS was demonstrated to exert a mood-enhancing effect in depressive patients, it remains unclear whether cognitive decrement in this population is similar to this in the general population (4, 5, 41, 42). In a project by Larson et al., individuals with clinical depression exhibited similar cognitive impairment to euthymic controls after DS (14). However, mood changes were not assessed. The literature on the effects of acute sleep loss is highly diverse, depending on, among others, study protocol, inclusion criteria, etc. Another important aspect is that depression is a highly complex disorder, which might reflect itself in the diversity of studies’ outcomes (43). It is worth emphasizing, that in this study, participants were not diagnosed with depression, MADRS was used only to evaluate mood changes.

It was also observed that the RE group performed better at PVT in the PSG morning than in the preceding evening, whereas scores obtained by NR individuals remained similar. One of the possible mechanisms behind enhanced vigilance after a night’s rest is the domination of the morning chronotype in this group. Chronotype is an individual trait that has a major impact on cognitive performance. According to one study individuals with morning chronotype performed on PVT similarly in the morning and in the evening, whereas those with evening chronotype had worse results at their non-optimal time, i.e., in the morning (44).

This relationship between chronotype, depression symptoms, and response to DS appears to be important in the context of future studies with depressive patients. Symptoms of this disorder show a diurnal pattern, usually being worse in the morning (45). Bouhuys et al. noticed an association between diurnal variation of symptoms and response to sleep deprivation, suggesting, that it might be one of the factors decisive in response to DS (46). A recent meta-analysis by He et al. demonstrated that assuming the chronobiological approach to sleep deprivation by implementing together DS and bright light therapy yields better results than sleep loss alone (42).

This study possesses several characteristics which distinguish it from the previous ones. Many projects conducted up to date used a limited battery of tests; psychomotor abilities, like eye-hand coordination appear to be particularly poorly researched (11). Here, due to the application of a range of tasks involving distinctive cognitive abilities (Stroop test, TMT, PVT), it was possible to comprehensibly assess alterations associated with sleep loss as well as notice certain patterns in results, like speed-accuracy trade-off. The present study is also one of the few that conducted a baseline assessment under the control of PSG, enabling the exclusion of individuals with sleep disorders, like insomnia. Lu et al. in a similar project also utilized PSG, however, they focused on electroencephalographic findings in depressed participants, rather than the elimination of those suffering from sleep disturbances (13). The majority of studies on the influence of DS on mood use scales other than MADRS; studies involving depressed patients tend to apply different versions of Hamilton depression rating scale, whereas those conducted on healthy subjects frequently utilize the Visual Analog Scale or State–Trait Anxiety Inventory (8, 47). The use of MADRS instead of other questionnaires allowed for addressing primarily depressive symptoms relating to mood, rather than assessing the somatic ones - focus was placed on reliable assessment of the state of mind, not reaching a psychiatric diagnosis. We also assumed more relaxed criteria for positive response to DS in comparison to other researchers, which increased the sensitivity to changes that occurred due to sleep loss (47, 48). Another important characteristic of this study is the high number of participants, which in other projects usually oscillated around 20–30 (11, 20).

This study has several limitations. One of them is the use of tests prone to the learning effect, that is BEHCT, TMT, and Stroop test. Application of other versions of those tasks would provide a more accurate comparison between baseline and post DS parameters. Assessment of other important cognitive functions, like working memory, was also omitted. Data analysis did not include information about participants’ chronotypes, which could aid in explaining differences in baseline evening/morning task results. Controlling participants during DS with actigraphy might be a more objective method than direct supervision, however this approach is not impermeable to errors, i.e., short naps might not be detected (23, 25, 27).

To summarize, respondents to sleep deprivation maintain or improve cognitive and psychomotor functions after acute sleep loss, whereas the performance of non-respondents is compromised. Alterations in vigilance appear to be particularly related to differences in the severity of depression symptoms. Future studies might focus on the investigation of differences between respondents and non-respondents depending on the DS protocol and depression severity. Projects exploring the functional and molecular mechanisms of reaction to sleep loss are also validated.

## Data availability statement

The raw data supporting the conclusions of this article will be made available by the authors, without undue reservation.

## Ethics statement

The studies involving humans were approved by the Bioethical Committee of the Medical University of Lodz (number: RNN/302/20/KE). The studies were conducted in accordance with the local legislation and institutional requirements. The participants provided their written informed consent to participate in this study.

## Author contributions

MS: Conceptualization, Formal analysis, Investigation, Methodology, Writing – original draft, Writing – review & editing. MD: Methodology, Writing – original draft. PB: Methodology, Writing – review & editing. ST: Methodology, Writing – review & editing. FK: Methodology, Writing – review & editing. AG: Conceptualization, Methodology, Supervision, Writing – review & editing.
