# Correction: Evaluation of cognitive and psychomotor faculties in relation to mood-related symptoms under the conditions of sleep deprivation

**DOI:** 10.3389/fpsyt.2025.1627775

**Published:** 2025-07-10

**Authors:** Marcin Sochal, Marta Ditmer, Piotr Białasiewicz, Szymon Turkiewicz, Filip Franciszek Karuga, Agata Gabryelska

**Affiliations:** Department of Sleep Medicine and Metabolic Disorders, Medical University of Lodz, Lodz, Poland

**Keywords:** sleep deprivation, PVT, hand-eye coordination, stroop, sleep

In the published article, there was an error in **Table 1** as published. The values ​​of the variable defining the number of women in the subgroups were presented incorrectly. The corrected **Table 1** appears below.

**Table 1:** Baseline characteristics of study participants.

Parameter	Non-respondents	Respondents	*P*
*n*	29	48	–
Women, *n*, %	15, 51.70%	23, 47.90%	0.883
Age, median (IQR)	23 (22–26)	24 (22–26)	0.541
BMI, [kg/m2], median (IQR)	22.05 (19.49–23.78)	22.85 (22.04–24.72)	0.065
Smoking, *n*, %	3, 10.30%	6, 12.50%	0.775
Higher Education, *n*, %	14, 48.30%	23, 47.90%	0.735
Total Sleep Time during the PSG night, [min], median (IQR)	400 (360–480.5)	430 (366–468.75)	0.987
Sleep Latency during the PSG night, [min], median (IQR)	33.5 (17.5–51)	34.5 (23.75–57.75)	0.467
Sleep efficiency during the PSG night [%], median (IQR)	78.6 (71.7–89.5)	81 (69.25–85.65)	0.487

BMI, Body Mass Index, PSG, polysomnography.

Results were presented as median (Interquartile range, IQR) or *n*, %.

The original article has been updated.

